# Taking Cold

**Published:** 1876-12

**Authors:** 


### TAKING COLD.

A large number of fatal winter diseases result from taking cold, and often from such slight causes, apparently, as to appear incredible to many. But, although the causes are various, the result is the same, and arises from the violation of a single principle, to-wit, cooling off too soon after exercise. Perhaps this may be more practically instructive if individual instances are named, which, in the opinion of those subsequently seeking advice in the various stages of consumption, were the causes of the great misfortune, premising that when a cold is once taken, marvellously slight causes serve to increase it for the first few days, causes which, under ordinary circumstances, even a moderately healthful system would have easily warded off.

Rachel the tragedienne, increased the cold which ended her life, by insufficient clothing in the cars, in travelling from New York to Boston; such was her own statement.

The immediate cause of the last illness of Abbott Laurence, the financier, and the philanthropist, was an injudicious change of clothing.

An eminent clergyman got into a cold bed in mid-winter within fifteen minutes after preaching an earnest discoure, he was instantly chilled, and died within forty-eight hours.

A promising young teacher walked two miles for exercise, and on returning to his room, it being considered too late to light a fire, sat for half an hour reading a book, and before he knew it, a chill passed over him. The next day he had spitting of blood, which was the beginning of the end.

A most promising and active clerk in a "Russia house" of great wealth in New York, was caught in a rain in going to the store, where he found a dozen or more letters which had to be answered by the steamer which was to start at noon. Being in most robust health, never having been sick an hour for years, he thought he would "get through with his letters first," before removing his dampened garments, as it was in mid-summer. Next day he had a hemorrhage of nearly a quart of blood, and at the end of four months he is scarcely able to "crawl out of the house."

A lady walked from "Stewart's" to Union Square on a beautiful spring day. On reaching home, she immediately changed her dress, in a room where there was no fire. The same night she was surprised by an attacked of asthma, which very nearly proved fatal within a week.

A mother sat sewing for her children to a late hour in the night, and noticing that the fire had gone out, she concluded to retire to bed at once; but thinking that she could "finish" in a few minutes, she forgot the passing time, until an hour more had passed, and she found herself "thoroughly chilled," and a month's illness followed to pay for that one hour.

Many a cold, cough and consumption is excited into action by pulling off the hat or overcoat as to men, and the bonnet and shawl as to women, immediately on entering the house in winter, after a walk. An interval of at least five or ten minutes should be allowed, for however warm or "close" the apartment may appear on first entering, it will seem much less so at the end of five minutes, if the outer garments remain as they were before entering. Any one who judiciously uses this observation, will find a multifold reward in the course of a lifetime.

